# Native Tissue Posterior Compartment Repair for Isolated Posterior Vaginal Prolapse: Anatomical and Functional Outcomes

**DOI:** 10.3390/medicina58091152

**Published:** 2022-08-25

**Authors:** Giuseppe Marino, Matteo Frigerio, Marta Barba, Tomaso Melocchi, Desirèe De Vicari, Andrea Braga, Maurizio Serati, Umberto Leone Roberti Maggiore, Alessandro Ferdinando Ruffolo, Stefano Salvatore, Stefano Uccella, Mattia Dominoni, Marco Torella

**Affiliations:** 1 Department of Obstetrics and Gynecology, Milano-Bicocca University, 20900 Monza, Italy; 2 ASST Monza, 20900 Monza, Italy; 3 EOC-Beata Vergine Hospital, 6850 Mendrisio, Switzerland; 4 ASST Sette Laghi, 21100 Varese, Italy; 5 Fondazione IRCCS Istituto Nazionale Dei Tumori, 20133 Milan, Italy; 6 San Raffaele Hospital, 20132 Milano, Italy; 7 AOUI Verona, University of Verona, 37129 Verona, Italy; 8 IRCCS Policlinico San Matteo, 27100 Pavia, Italy; 9 Department of Obstetrics and Gynecology, University of Campania “Luigi Vanvitelli”, 80138 Napoli, Italy

**Keywords:** pelvic organ prolapse, posterior compartment, native-tissue repair, obstructed defecation syndrome, constipation

## Abstract

*Background and Objectives:* Posterior compartment prolapse is associated with constipation and obstructed defecation syndrome. However, there is still a lack of consensus on the optimal treatment for this condition. We aim to investigate functional, anatomical, and quality-of-life outcomes of native tissue transvaginal repair of isolated symptomatic rectocele. *Materials and Methods:* We retrospective analyzed patients who underwent transvaginal native tissue repair for stage ≥ II and symptomatic posterior vaginal wall prolapse between January 2018 and June 2021. Anatomical and functional outcomes were evaluated. Wexner constipation score was used to assess bowel symptoms, while the Patient Global Impression of Improvement (PGI-I) score was used to evaluate subjective satisfaction after surgery. *Results:* Twenty-eight patients were included in the analysis. The median age was 64.5 years, and half of them underwent a previous hysterectomy for benign reasons. The median follow-up time was 33.5 months. A significant anatomical improvement in the posterior compartment was noticed compared with preoperative assessment (*p* < 0.001 for Ap and Bp), with only two (7.1%) anatomical recurrences. Additionally, obstructed defecation symptoms decreased significantly compared to baseline (*p* < 0.001), as well as vaginal bulging, with no new-onset cases of fecal incontinence or de novo dyspareunia. PGI-I resulted in 89.2% of patients being satisfied (PGI-I ≥ 2), with a median score of 1.5. *Conclusions:* Transvaginal native tissue repair for isolated posterior prolapse is safe and effective in managing bowel symptoms, with excellent anatomical and functional outcomes and satisfactory improvement in patients’ quality of life.

## 1. Introduction

Pelvic organ prolapse (POP), also called urogenital prolapse, is defined as the descent of the uterus, bladder, rectum, and/or bowel throughout the vagina [1]. POP is a common clinical condition, affecting 43–76% of parous women [2]. Risk factors for the development and recurrence of prolapse include obstetric factors, chronic increased abdominal pressure, and altered collagenic patterns [3,4,5]. Symptoms may depend on the involved compartment and include bulging symptoms, urinary storage, voiding function, and obstructed defecation [6]. While the POP typical presentation is a multicompartment combination of anterior, central, and posterior defects, single-compartment herniation is also described [1]. In particular, isolated rectocele—or posterior vaginal wall prolapse—is a more unusual entity involving the herniation of the anterior rectal wall that produces a posterior vaginal bulge, generally due to a defect or a lack of rectovaginal septum [7,8]. This may be totally asymptomatic or associated with a wide range of symptoms, including lump/sensation of vaginal fullness and alteration in bowel and sexual functions [9]. In particular, constipation and obstructed defecation syndrome are often reported, and in most severe cases, there is a need to digitally reduce prolapse or perform manual evacuation to remove the stool [10]. Depending on the severity of the prolapse and the symptoms, the first approach must be based on conservative management, including changes in diet, keeping stool soft, also using oral or rectal drugs, and pelvic floor muscle training. In case of failure of the conservative management, surgical repair represents the gold standard and is aimed to restore both anatomy and bowel function. However, while it is well established that prolapse surgery is efficacy in reducing bulging symptoms, bladder storage, and voiding symptoms, there are only few data on functional outcomes of isolated rectocele repair [11,12,13,14]. Moreover, since both constipation and obstructed defecation are multifactorial conditions, there is uncertainty if posterior compartment anatomical restoration can be effective in improving bowel function.

Consequently, with this study, we aimed to investigate functional, anatomical, and quality-of-life outcomes of native tissue transvaginal repair of isolated symptomatic rectocele.

## 2. Materials and Methods

Between January 2018 and June 2021, patients who underwent transvaginal native tissue repair for stage ≥ II and symptomatic posterior vaginal wall prolapse were retrospectively analyzed. Patients with incomplete data or with a follow-up of fewer than 12 months were not considered. Preoperative evaluation included a medical interview to assess obstetric history, age, body mass index, cigarette smoking, and menopausal status. The presence of symptoms was defined according to the International Urogynecology Association and International Continence Society standardization of terminology [15]. Wexner score was used to evaluate bowel dysfunction severity [16]. This test evaluates different aspects of defecation (frequency of defecation, difficulty, incompleteness, duration of defecation, unsuccessful attempts, abdominal symptoms, and duration of the problem, regardless of the administration of laxatives). For each of the steps, there is a score from 0 to 4 points, while as regards the assistance to defecation, from 0 to 2 points are foreseen. It’s defined as mild (1–5), moderate (6–10), severe (11–15), and very severe constipation (16–30) on the basis of the total score. A urogenital examination was performed, and POP was staged according to the Pelvic Organ Prolapse Quantification system (POP-Q) [17]. Patients underwent transvaginal native tissue posterior compartment repair according to the described technique [18]. An inverted-T posterior colpotomy extending from the vaginal apex to the hymen was performed. Wide dissection of the posterior vaginal wall from the rectovaginal tissue was carried out to isolate rectal prolapse, and half-purse string sutures reduction was performed. In the case of Douglas pouch opening during the procedures, isolation and excision of enterocele sac and pouch of Douglas obliteration with a purse-string suture were performed. The puborectalis muscle was identified lateral to the rectum and distal from the ischial spines. Under direct vision, midline plication sutures were placed on the most medial portion of the puborectalis muscles on each side. Perineoplasty and posterior colporrhaphy completed the procedure. All the procedures were performed by experienced pelvic floor surgeons. Patients were followed up 1 month and 1 year after surgery, and then yearly. Follow-up visits included a clinical interview and a complete urogenital examination. Wexner’s score was used to evaluate postoperative constipation. The postoperative presence of bulging symptoms was considered a subjective recurrence. A postoperative descent to stage II or below according to the POP-Q system in the posterior compartment or the need for reoperation was considered as objective recurrence. The Patient Global Impression of Improvement (PGI-I) score was used to evaluate subjective satisfaction after surgery [19]. QoL success was defined by both “very much improved” and “much improved” at the PGI-I score (score ≤ 2).

The study was approved by the Institutional Review Board of San Gerardo Hospital in Monza, Italy (SH-MCC 1709/2013). Data were collected from hospital-dedicated software for patients’ clinical monitoring. Data were entered into the database by one author and double-checked by one other author. Descriptive statistics were calculated as absolute numbers with percentages for categorical variables and as median (interquartile range) for continuous ones. Differences were tested using paired *t*-test for continuous data and Fisher’s test for noncontinuous data. A *p*-value < 0.05 was considered statistically significant.

## 3. Results

A total of 30 patients underwent transvaginal native tissue posterior compartment for an isolated rectocele in the period of interest. Two of them were excluded from the analysis due to missing data/loss at follow-up. As a consequence, 28 patients were evaluated for the analysis. Populations’ characteristics are described in Table 1. The median age was 64.5 years, with 92.9% of patients being in menopausal status. Half of them had a previous hysterectomy for prolapse or other benign indications (eight vaginal hysterectomies, five open abdominal hysterectomies, and two laparoscopic hysterectomies). Baseline symptoms and prolapse stage and POP-Q are shown in Table 2. All women had a posterior compartment prolapse stage II or above. All of them presented bowel disorders: 25 (89.3%) women referred obstructed defecation (with or without the need to digitally reduce prolapse or acting manual evacuation/digitation), while 3 (10.7%) were bothered by anal incontinence. Moreover, 24 (85.7%) of women referred vaginal bulging symptoms, and 19% of sexually active women were bothered by dyspareunia. All women underwent isolated transvaginal native tissue repair of the posterior compartment. The procedures were completed in 30.0 (26.0–35.0) minutes of median operative time, with a median blood loss of 100.0 (100.0–100.0) mL. No intraoperative complications were observed. One (3.6%) postoperative complication occurred. This was a rectovaginal space hematoma associated with severe pain observed on postoperative day 1 that was managed with reintervention for surgical drainage. No long-term complications (>30 days from surgery) were observed. The median follow-up time was 33.5 (24.0–48.0) months. Two (7.1%) anatomical recurrences were observed. One of them (3.6%) was symptomatic and required reintervention, while the other one was asymptomatic. Consequently, objective and subjective cure rates were 92.9% and 96.4%, respectively. A comparison between preoperative and postoperative posterior vaginal profiles according to the POP-Q system is shown in Table 3. A significant improvement in the posterior compartment according to the POP-Q system was noticed compared with preoperative assessment (*p* < 0.001 for Ap and Bp), with a concomitant increase in pb (*p* < 0.001) and reduction in gh (*p* < 0.001). Functional outcomes are reported in Table 4. Obstructed defecation symptoms decreased significantly compared to baseline (*p* < 0.001), as well as vaginal bulging. Interestingly, no new-onset cases of fecal incontinence or de novo dyspareunia were observed. Wexner’s constipation scale showed a significant improvement compared to baseline, scoring a median of 7 points less after surgery (from 10 to 3). Quality-of-life outcomes evaluated with PGI-I resulted in 89.2% of patients being satisfied (PGI-I ≥ 2), with a median score of 1.5 (interquartile range 1.0–2.0).

## 4. Discussion

The impact of surgical repair on functional outcomes in patients with posterior prolapse and bowel dysfunction is still unclear. Moreover, a wide variety of surgical interventions and approaches exist for the management of posterior vaginal wall prolapse, and there is still a lack of consensus on the optimal treatment, thus generating confusion and bias. While recent data have shown persisting and favorable functional outcomes for transvaginal native tissue repair for pelvic organ prolapse at long follow-up [20], there is still a lack of data on the efficacy of this kind of surgery for isolated posterior compartment prolapse correction.

With this study, we aimed to investigate whether transvaginal native tissue repair is effective in terms of outcomes, with particular respect to functional ones. We found that surgical repair of isolated posterior compartment prolapse is safe and effective in ameliorating obstructed defecation and bulging symptoms without introducing de novo dysfunctions. In particular, obstructed defecation symptoms drastically decreased from 89.3% at baseline to 3.6% after surgery. Concomitantly, Wexner’s constipation scale scores decreased by a median of 7 points for the effect of the surgery. These findings demonstrated that native tissue posterior compartment repair is able to recover most obstructed defecation symptoms in patients with isolated posterior compartment prolapse. This is consistent with a previous retrospective study by Schiavi in a cohort of 151 patients who underwent transvaginal native tissue repair for posterior compartment prolapse [21]. Concomitant procedures such as hysterectomy, high uterosacral suspension, anterior colporrhaphy, and anti-incontinence surgery were performed in 20.2%, 23.2%, 29.8%, and 20.5% of patients, respectively. They found a significant decrease in defecatory dysfunctions (from 30.5% to 3.3%), vaginal digitation (from 14.6% to 0%), and vaginal bulge (from 24.5% to 1.3%) after the surgical correction. From an anatomical point of view, we recorded an improvement in gh, pb, Ap, Bp, and D points, with a minimal objective recurrence (7.1%) and reoperation rate (3.6%). Our data are similar to one reported by Schiavi, in which at one year, only 11.3% of patients experienced posterior relapse higher than II stage according to the POP-Q system [21]. Moreover, we found a very low complication rate (3.6%), with de novo dysfunction arising after surgery, including anal incontinence and dyspareunia. This confirms that transvaginal native tissue surgery for posterior compartment prolapse is a safe procedure. The high efficacy and minimal short- and long-term complication may explain the high satisfaction rate that we found in our series, with 89.2% of patients defining themselves as “improved” or “very improved” according to the PGI -I score.

As a consequence, our experience confirms that transvaginal native tissue repair may be particularly adequate for treating patients with symptomatic isolated posterior compartment prolapse [22,23]. However, different surgical techniques have been described for the management of this condition, including the implantation of mesh materials. However, comparison studies demonstrated a rate of mesh exposure up to 7%, with no substantial benefits in the use of prosthetics in the posterior compartment [24,25]. For these reasons, the Cochrane review has concluded that evidence does not support the utilization of mesh materials at the time of posterior compartment correction for isolated posterior compartment prolapse [22]. Moreover, transvaginal repair has been demonstrated to be superior to the transanal approach in terms of subjective and objective outcomes [20,22,26,27,28]. Recently, a systematic review on surgical interventions for posterior compartment prolapse and obstructed defecation symptoms was published, trying to untangle this thorny question about choosing the better surgical way to treat posterior compartment prolapse [29]. This paper compared the impact of several surgical interventions, including native-tissue transvaginal rectocele repair, transanal rectocele repair, stapled transanal rectocele repair surgeries, and sacrocolpoperineopexy. Based on anatomical and functional outcomes and complications, the authors concluded that in these patients, a native-tissue transvaginal rectocele repair should be preferentially performed [29].

To the best of our knowledge, this is the first study focusing on anatomical, functional, and quality-of-life outcomes using validated tools—such as Wexner and PGI-I scores—in patients who underwent isolated transvaginal native tissue repair of the posterior compartment for symptomatic rectocele. In addition, patients were evaluated after a substantial follow-up time. This study added to the current state of the art the evidence that isolated native tissue repair of the posterior compartment is associated with anatomical, functional, and quality-of-life improvements. Limitations include the retrospective design and the limited number of patients analyzed. However, we decided to evaluate only patients who had isolated posterior compartment repair in order to reduce confounding factors and possible sources of bias and obtain a homogeneous population.

## 5. Conclusions

In conclusion, transvaginal native tissue repair for isolated posterior prolapse is safe and effective in managing bowel symptoms. Moreover, surgical management is associated with excellent anatomical outcomes with satisfactory improvement in patients’ quality of life.

## Figures and Tables

**Table 1:** Population characteristics. Data as absolute (relative) frequencies for categorical variables and median (interquartile range) for continuous ones.

Age (Years)	64.5 (55.3/76.5)
BMI (kg/m^2^)	26.3 (24.9/27.3)
Cigarette smoking	5 (17.9%)
Parity (n)	2.0 (2.0–2.0)
Operative delivery	1 (3.6%)
Menopausal status	26 (92.9%)
Previous hysterectomy	14 (50%)

**Table 2:** Preoperative symptoms and prolapse stage. Data as absolute (relative) frequencies for categorical variables and median (interquartile range) for continuous ones; * only for sexually active patients; ** only for patients with uterus.

Bulging Symptoms	24 (85.7%)
Sexual activity	21 (75.0%)
Dyspareunia *	4 (19.0%)
Anal incontinence	3 (10.7%)
Obstructed defecation	25 (89.3%)
Wexner score	10 (8.3/12.0)
Posterior compartment POP stage	3.0 (2.0/3.0)
Aa	−2.8 (−3.0/−2.0)
Ba	−2.8 (−3.0/−2.0)
C	−8.0 (−8.0/−6.3)
gh	4.0 (3.0/4.0)
pb	2.3 (2.0/3.0)
tvl	9.0 (8.0/10.0)
Ap	1.5 (0.0/2.0)
Bp	1.5 (0.0/2.0)
D **	−4.5 (−6.3/−4.0)

**Table 3:** Preoperative versus postoperative POP-Q comparison. * only for patients with uterus.

	Preoperative	Postoperative	*p* Value
Aa	−2.8 (−3.0/−2.0)	−2.8 (−3.0/−2.0)	0.764
Ba	−2.8 (−3.0/−2.0)	−2.8 (−3.0/−2.0)	0.764
C	−8.0 (−8.0/−6.3)	−8.0 (−8.0/−7.0)	1.000
gh	4.0 (3.0/4.0)	3.0 (3.0/4.0)	<0.001
pb	2.3 (2.0/3.0)	3.0 (3.0/3.5)	<0.001
tvl	9.0 (8.0/10.0)	9.0 (8.3/10.0)	0.746
Ap	1.5 (0.0/2.0)	−3.0 (−3.0/−2.0)	<0.001
Bp	1.5 (0.0/2.0)	−3.0 (−3.0/−2.0)	<0.001
D *	−4.5 (−6.3/−4.0)	−7.0 (−7.0/−5.0)	0.024

**Table 4:** Functional outcomes. Data as absolute (relative) frequencies for categorical variables and median (interquartile range) for continuous ones. * only for sexually active patients.

	Preoperative	Postoperative	*p* Value
Bulging symptoms	24 (85.7%)	1 (3.6%)	<0.001
Sexual activity	21 (75.0%)	21 (75.0%)	1.000
Dyspareunia *	4 (19.0%)	1 (4.8%)	0.343
Anal incontinence	3 (10.7%)	0 (0%)	0.236
Obstructed defecation	25 (89.3%)	1 (3.6%)	<0.001
Wexner score	10.0 (8.3/12.0)	3.0 (2.0/4.0)	<0.001

## Data Availability

The data presented in this study are available on request from the corresponding author. The data are not publicly available due to privacy policy.
